# Fatigue Life Assessment of Rolling Bearings Made from AISI 52100 Bearing Steel

**DOI:** 10.3390/ma12030371

**Published:** 2019-01-24

**Authors:** Paweł J. Romanowicz, Bogdan Szybiński

**Affiliations:** Institute of Machine Design, Cracow University of Technology, ul. Warszawska 24, 31-155 Cracow, Poland; boszyb@mech.pk.edu.pl

**Keywords:** rolling bearings, fatigue, multiaxial criteria, size effect, contact problems

## Abstract

The rolling bearings used in various industrial applications are exposed to fatigue failure during their operation. Generally, in a practical application, the ISO 281:2007 standard is used for fatigue life assessments of rolling bearings. The application of the formula given in this standard requires knowledge concerning the basic dynamic load rating *C*. The natural question is raised of whether it is possible to omit the time-consuming experimental tests while still effectively calculating the fatigue load or the load capacity of the bearings. In the paper, the authors propose the application of analytical formulas for stresses in the contact area and its vicinity, and the usage of the multiaxial high-cycle fatigue hypothesis to estimate the maximal fatigue load for the rolling bearings. In the proposed methodology, only the knowledge concerning the fatigue properties of the material and geometry characteristics of the analyzed bearings are demanded. The effectiveness of the authors’ proposal is verified for arbitrarily chosen bearings. The observed discrepancy between the catalogue fatigue load (SKF catalogue) and numerically calculated fatigue load usually does not exceed 10%, which is fully acceptable from an engineering point of view and justifies the approach proposed in the paper. The proposed methodology can be used for the prediction of the fatigue life and optimization of the rolling bearings.

## 1. Introduction

Rolling bearings are commonly and widely used subassemblies in rotary machinery systems (RMS). They are used in the bearing arrangements of small and precise devices as well as in large industrial machines. The elements of bearings, such as balls, rollers, or raceways are exposed for multiaxial and non-proportional low and high-cycle fatigue loadings, which are usually the source of the rolling contact fatigue (RCF) [1,2,3,4,5]. This kind of damage is the dominant form of failure for rolling bearings. The fatigue life of bearings depends on many factors and variables, such as the size and direction of the loadings, the geometry and roughness of the contact surfaces, the kind of material, the operating temperature, applied lubricant, lubrication conditions, rolling speed, and sliding in the contact area [5,6,7]. Due to the small region of the contact area, the resulting stresses are concentrated in a small subsurface volume. This results in a high concentration of contact and subsurface stresses, which can be the source of fatigue cracks. The initiation of them may begin either at surface defects [8] or under the contact surface [3,7,9,10,11,12,13]. The further propagation of fatigue cracks depends on different factors, such as the shear stress level, material structure (austenite/martensite), temperature [14], cyclic deformation, the presence of voids and defects, etc. The RCF process may be also influenced by the evolution of the microstructure, which may result in the appearance of white etching areas, cracks, and wear debris [2,7]. Several attempts have also been made to assess and understand crack propagation mechanisms [12,13].

As the possible damage of the rolling bearing determines the operation of the RMS, the proper estimation of the fatigue life or loading capacity of a rolling bearing becomes the crucial task in the machine design process. Additionally, difficulties with the detection of bearing element damages at the initial stages (damage detection is possible when damage achieves a certain size) justify the fatigue life prediction of rolling bearings.

As mentioned before, many factors have an influence on the fatigue strength of roller bearings. One of the main important parameters is the shape of the roller and the raceway profile [15,16,17] (in roller bearings) or association of ball and raceway radii (in ball bearings) [18]. Poplawski et al. [16] studied the influence of different shapes of roller profiles (flat, end-tapered crown, aerospace crown, and full crown) on fatigue life. It was reported that the highest fatigue strength can be obtained for end-tapered crown profiles [16]. These are related to the existence of a bigger contact area than for the other crown profiles. It is worth mentioning that the highest contact area, which exists in the case of a flat profile, does not provide the largest fatigue strength. This decrease of the fatigue life is attributed to the presence of the surface and subsurface stress concentrations located along the edges of the contact area [16]. Another parameter, which has a significant influence on the fatigue life, is the internal diameter clearance between rollers and races [19]. The existence of an incorrect interference fit between the shaft and the roller bearing may also induce tensile stresses in the bearing, and then reduce its fatigue life [6].

The research studies related to fatigue life prognostics and health monitoring for RMS have important meaning in both academia and industry. Lee et al. made a detailed review of the diagnostic methods [20]. It should be noted that the health monitoring methods are still under development. As an example, the health degradation monitoring and the early fault diagnosis based on complete ensemble empirical mode decomposition with adaptive noise and the improved multivariate multiscale sample entropy [21] or through using acoustic emission [22] can be mentioned.

The elements of bearings are subjected to the non-proportional multiaxial loading conditions with large three-dimensional compressive stresses [2,18,23]. Different algorithms and criteria for the prediction of rolling bearing fatigue life have been proposed so far [4,19,24]. Prediction of the fatigue life of rolling bearings can be alternatively made with the use of empirical or analytical models. Sadeghi et al. presented a detailed critical review of such life models [4]. Such engineering life prediction models can be divided into two groups: deterministic and probabilistic (empirical). Empirical formulations are in commonly used in engineering applications. Some of them have simple mathematical formulations, but require experimental fatigue tests. On the other hand, the deterministic models (i.e., multiaxial criteria, plastic strain accumulation hypotheses, dislocation dynamics, etc.) are more complicated than probabilistic models, and require sufficient knowledge about material parameters (i.e., σ-ε and σ-*N* characteristics). In common engineering applications, the selection process and rolling bearing calculations are determined by the ISO standard 281:2007 [25] based on Palmgren–Lundberg and Ioannides–Harris criteria [4]. In the proposed model [25], it is assumed that the elements of rolling bearings are made of AISI 52100 alloy steel (100Cr6), and the rated service life can be calculated using the formula:

(1) $$L=a_{1}\cdot a_{2}\cdot a_{3}\cdot {\left(\frac{C}{P}\right)}^{k},$$

where: *L*—rated service life (in millions of revolutions) for 10% probability of failure, *a*_1_—modification factor for reliability, *a*_2_—material and processing factor, *a*_3_—application factor, *C*—basic dynamic load rating [N], *P*—external equivalent load [N], and *k*—exponent of bearing type (*k* = 3 for ball bearing, *k* = 10/3 for roller bearing).

This above given simple empirical criterion requires experimental data for particular rolling bearings. Such experimental tests are time-consuming and expensive. Formula (1) becomes difficult to use in the case of a more complex histogram of bearing loads (variation of external forces or rotational speeds, breaks at bearing operation, etc.). Such a situation applies to the exploitation of the vehicle. In this case, the mileage of the vehicle determines the moment of bearing replacement, even if the bearing does not manifest the symptoms of wear. The model proposed in the ISO standard [25] was recently criticized due to the assumption of the fatigue limit for AISI 52100 alloy steel. In the ISO standard, the Von Mises stress of 900 MPa is assumed as the fatigue limit. Unfortunately, the experimental tests performed by Shimizu et al. [26] and Saki [27] denied this assumption. This naturally raises doubts regarding whether the commonly used formulas are still versatile for any chosen rolling bearing. A promising alternative for the fatigue life estimation of rolling bearings is the application of the multiaxial high-cycle fatigue criteria (MHCFC). Such hypotheses take into account the influence of the complex distribution of multiaxial and non-proportional stresses over time on the equivalent fatigue level. Romanowicz discussed the verification of the selected criteria and their application to the rolling contact problems [28,29]. It was observed that the hypothesis proposed by Papadopoulos et al. [30] remains in good agreement with the experimental tests performed for rolling contact fatigue [31]. The application of this criterion requires only the knowledge of the course of the Wöhler SN (cyclic stress versus the number of cycles) curve of bearing steel and the distributions of subsurface stresses in rolling elements. Such SN curves for AISI 52100 alloy steel were determined by Shimizu et al. [26] and Saki [27].

The subsurface stresses in a rolling element or raceway can be calculated using the mathematical formulas based on the Hertz theory for elliptical or line contact. In the case of ball bearings (the elliptical contact), the solution given by Sackfield and Hills [32] can be adopted, while in the case of roller bearings (the line contact), the solution presented by Radzimovsky can be used [33]. The validity of both models was verified with the use of finite element (FE) analysis [18,23,34]. It suggests that FE analysis can be successfully applied for a more complicated shape of a rolling element [1,15,29]. The above-mentioned theoretical models do not take into account the influence of residual stresses and the tangential traction on the distributions of subsurface stresses. Guo and Barkey studied the influence of residual stresses and tangential traction on RCF of elements made of AISI 52100 bearing steel numerically and experimentally [35]. On the basis of their research, two important conclusions can be formulated: the surface residual stresses and friction effects have a small influence on the subsurface RCF, and the residual stresses affect mainly the surface fatigue and near-surface damage initiation and crack growth [35,36]. Govindarajan and Gnanamoorthy also developed a distribution of contact stresses under rolling and sliding conditions for sintered and hardened steels [36]. Vrbka et al. carried out experimental studies of the influence of surface texturing on the RCF of elements working under lubrication conditions [37], and they did not observe a distinct reduction of fatigue life for textured surfaces. Due to the given above observations, in the analysis of subsurface crack initiation and propagation, the tangential load, residual stresses, and surface texturing may be omitted in the fatigue calculations. The location of the fatigue crack initiation and fatigue life mainly depend on the normal load and the contact area. These justify the application of elliptical and line contact analytical solutions [32,33] for calculations of subsurface stresses in the elements of rolling bearings.

Taking into account all of these aspects, the authors proposed the original procedure for rolling bearing fatigue life prediction. This procedure is based on the application of the MHCF hypothesis and analytical solutions for subsurface stress distributions. Such an approach brings the following advantages:

1. The procedure does not require time-consuming and expensive experimental tests,
2. The calculation can be quickly and easily made using theoretical or FE models,
3. It allows for the optimization of shapes of rolling elements and raceways.

The paper is divided into seven chapters. In the first chapter, an introduction concerning the state of the art of the fatigue assessment of rolling bearing is provided. In chapter 2, various aspects of fatigue appearing between rolling bearing elements remaining in contact are presented. The fatigue properties of the bearing steel AISI 52100 are included in chapter 3. The next chapter describes the basic information concerning the rolling bearing geometries. The proposed methodology for a fatigue analysis of rolling bearings is presented in chapter 5. The analytical procedures for the determination of the internal loadings in bearing elements are discussed in 5.1. In point 5.2, the selected formulas for calculations of contact stresses are given. The adopted multiaxial fatigue model is presented in point 5.3. In the model, the influence of the size effect was introduced, which is the original proposal of the authors. In the chapter 6, the results of the extended studies for various types of bearings are presented and discussed. The paper is concluded in chapter 7.

## 2. Fatigue in Rolling Bearings

The rolling bearing elements are subjected to non-proportional multiaxial and time-dependent loading conditions with the occurrence of high compressive stresses [3]. Such stress distribution is caused by the moving contact zone. Generally, the surface and subsurface stresses in the surroundings of the contact zone can be described by Hertz’s solution. In spite of the occurrence of high contact stresses (up to three GPa [38]), the plastic deformations at the contact surface are not present [39]. This is due to the existence of the three-dimensional compression stress state, in which the differences between the principal stresses are small. The maximal shear stress at the surface is about 0.4*p_o_* [3] (*p_o_* is the maximal contact pressure).

Generally, under rolling contact loading conditions, the following forms of fatigue cracks can be distinguished:

- surface cracks (pitting)—triggered by surface defects or roughness and the presence of micro-notches, through which further crack growth can be accelerated by grease penetration [40],
- subsurface cracks (spalling)—caused by the alternate shear stresses, the presence of inclusions, etc., which originate cracks near the surface of rolling [3],
- cracks originated at deep defects also appearing in the low-stress zone.

In properly installed and lubricated bearings, the propagation of subsurface spalling is the main form of fatigue failure. Microcracks initiate below the surface (i.e., around inclusion, in high-stress volume, etc.) and grow into the surface. The inclusions can act in the same way as notches. It is observed that inclusions with sizes larger than 13 μm significantly decrease the fatigue life [39,41,42]. The definition of the critical size and shape of inclusion, which leads to the crack initiation, is more complicated and depends on the applied load, material structure, etc. Generally, such a mechanism leads to high-cycle fatigue. In such a regime, the crack initiation and propagation is caused by shear effects [10,43]. On the other hand, surface pitting is usually initiated by surface defects or non-ideal operating conditions, and is related to a high friction appearance. High stresses and the poor finishing of contact surfaces may lead to the failure in a regime of low-cycle fatigue [44]. The presence of such imperfections results in microcyclic plasticity [45], and may also lead to the ratcheting phenomenon. It means that the plastics zones are very limited, and the structure at the macro scale still remains in an elastic state. Such failure mechanisms are also observed in other applications in which contact stresses occur, such as gears, railway or crane wheels, rails, or cam–tappet mechanisms.

The amplitude of stresses plays a significant role in fatigue crack growth. Two special cases of the contact usually occur in practical applications:

- steady-state contact (lack of rolling or rolling with low velocity) under various loading, and
- rolling contact (RC).

**Figure 1:**
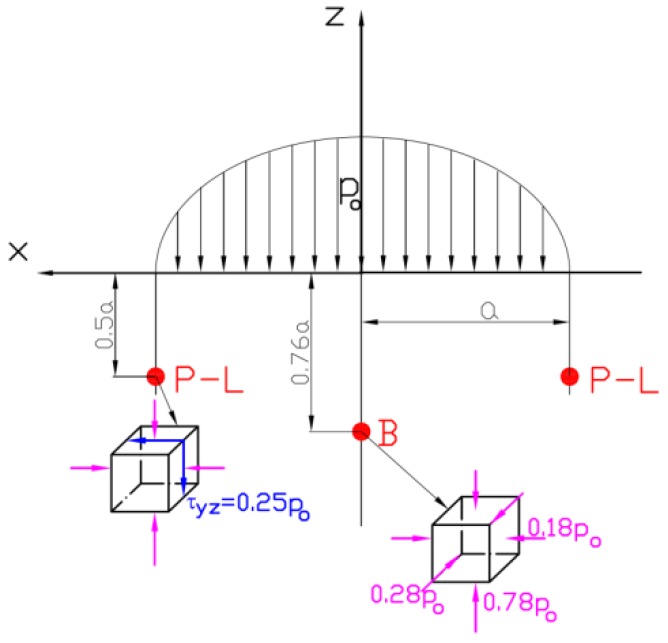
Location of characteristic points under surface, P-L—Palmgren–Lundberg points, B—Bielajev point.

**Figure 2:**
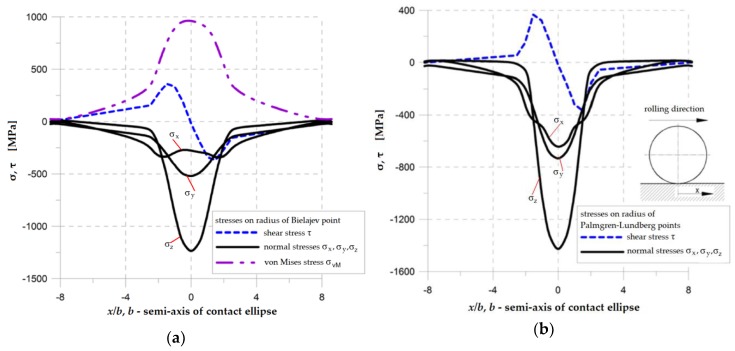
Distributions of normal and shear subsurface stresses for rolling bearing 6204* (loading *Q_max_* = 153 N, *f* = 0.544, *f* is defined in Equation (5)) (**a**) on radius corresponding to Bielajev point; (**b**) on radius corresponding to P-L points.

**Figure 3:**
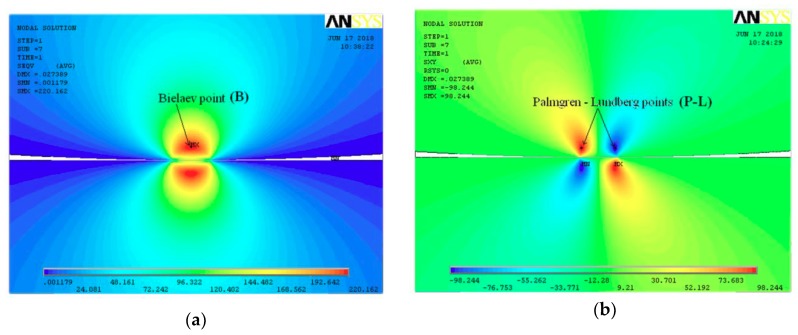
Distribution of subsurface stresses in rolling element (obtained by finite element method (FEM)): (**a**) von Mises stress; (**b**) shear stress.

In the first case, the amplitude of the maximal equivalent stress, which appears in the Bielajev point (Figure 1, Figure 2a and Figure 3a), can play the dominant role in fatigue crack nucleation. In the second case, the fatigue cracks are generally initiated in close vicinity to the rolling surface and are caused by the amplitude of shear stresses (Figure 1, Figure 2b and Figure 3b). A typical subsurface stress distribution for the RC case is presented in Figure 2. It can be observed that during rolling, the shear stresses change their direction (see Palmgren–Lundberg’s points in Figure 1, Figure 2b and Figure 3b). TThe characteristic feature is that the normal compressive stresses are shift in-phase with respect to shear stresses. It has been experimentally proven that this effect causes a reduction of the fatigue life for hard materials (such as bearing steels) [31]. It was also observed that critical planes (in which the maximal shear stress occurs) change their orientation during rolling [28]. A rotation of the critical plane results in a decrease of the fatigue life for hard materials [46,47]. The detailed study of subsurface stress distribution, analysis of the locations of critical points, and determination of the critical planes in which cracks may be initiated are discussed in Ref. [28].

## 3. Material

The elements of the rolling bearings are generally made of the bearing steels that usually contain 0.8–1.1% C and less than 3% of the alloy additives (such as Cr, Mn, Si, Ni, Mo, and Cu) [38]. AISI 52100 bearing steel (100Cr6 in DIN and EN-ISO Standards) is the most often used in the manufacturing of bearing elements. The chemical composition of AISO 52100 is given in Table 1. This material has excellent corrosion resistance, good wear resistance, and high compressive strength (the yield limit is 2000 MPa, and the tensile strength after heat treatment is 2250 MPa [8]). AISI 52100 is also adopted in the mathematical model for the calculations of rolling bearings that were proposed in ISO Standard 281:2007 [25]. In the model [25], the fatigue limit was associated with the von Mises stress *σ_vM_* and equal to *σ_vM_* = 0.9 GPa. This value of the von Mises stress is corresponding to the maximal contact stress 1.5 GPa.

The fatigue limit (*σ_vM_* = 900 MPa) assumed in the Standards [25] was recently criticized and questioned [26,27]. Shimizu et al. [26] and Saki [27] also experimentally observed that heat-treated AISI 52100 bearing steel does not have an observable fatigue limit.

**Figure 4:**
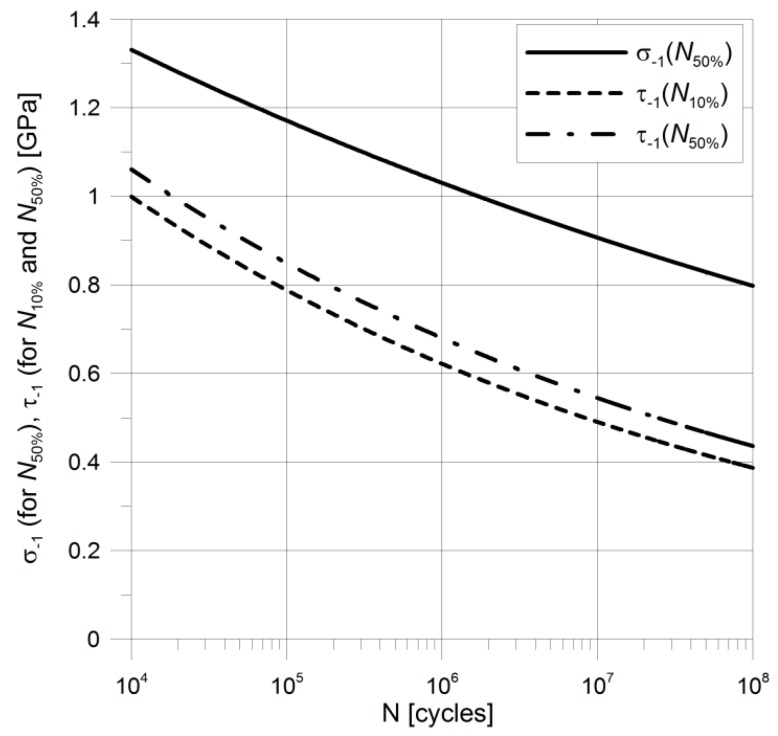
SN curves for AISI 52100 in alternating axial and alternating torsion loading for 10% failure life (*N_10%_*) and 50% failure life (*N_50%_*).

In order to estimate the fatigue life or the maximal capacity of the roller bearing, it is necessary to use the SN curves of the material (Figure 4). The results of the experimental tests for alternating axial and alternating torsion loadings [26,27] show that the AISI 52100 (60HRC) bearing steel does not have a clear fatigue limit (Figure 4). In such case, the fatigue limits for a particular number of hoisting cycles for AISI 52100 can be estimated using the given below formula:

- for median life (50% of failure life):
  (2) $$\sigma_{-1}\;(GPa)=2.22\cdot N_{50\%}{}^{-0.0556},$$
  (3) $$\tau_{-1}\;(GPa)=2.58\cdot N_{50\%}{}^{-0.0965},$$
- for 10% failure life (90% rating life for material):
  (4) $$\tau_{-1}\;(GPa)=2.58\cdot N_{10\%}{}^{-0.103}.$$

## 4. Rolling Bearing Geometry

**Figure 5:**
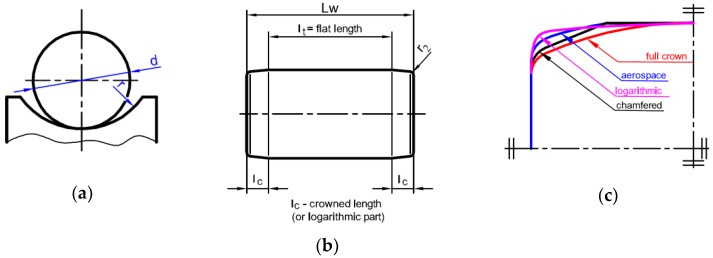
Geometry of rolling element: (**a**) ball and race conformity; (**b**) typical shape of cylindrical roller; (**c**) other shapes of cylindrical rollers.

The roller geometry and the race conformity are the main parameters affecting the fatigue strength of the ball bearings. The race conformity is defined as an adjustment of the geometrical shape of the ball and the race in a plane passing through the bearing axis using the formula:

(5) $$f=\frac{r}{d},$$

where *r* and *d* are defined in Figure 5a.

Roller bearings are subjected to higher loadings than ball bearings. Cylindrical rollers are also more sensitive for edge contact stresses [16,34] and misalignment. To avoid or to minimize these effects, certain modifications of rollers were proposed. The cylindrical rollers are chamfered, partially or full-crowned, etc. (Figure 5c). The influence of the roller shape was discussed in Refs [15,16]. The typical shape of the cylindrical roller is presented in Figure 5b. Generally, three parts of roller shape can be distinguished: a central flat part with length *l_t_*, and two crowned parts, with lengths *l_c_* on both sides of a roller. The *r*_2_ values of the fillet radius of the edges of a roller are given in Standards [48].

## 5. Analytical Procedure for the Fatigue Life Assessment of Rolling Bearings

Fatigue life analysis requires a prior determination of the distributions of stresses as functions of time. It can be made using a numerical method (such as the finite element method) or theoretical solutions. The application of numerical techniques requires the preparation of numerical models, meshes, boundary conditions, etc. This process is time-consuming. So, if it is possible, the application of the theoretical solutions in practical engineering cases provides clear advantages, and is preferred. As an example, the distribution of subsurface stresses in rolling elements of roller and ball bearings can be calculated using theoretical approaches. Historical but also essential solutions for non-frictional elastic contact were given by Hertz in 1882 [49]. The assumptions and formula proposed by Hertz were intensively developed for different contact cases [32,33,36,50]. The most important in RCF analyses are solutions for:

- Subsurface stresses for elliptical contact [32],
- Subsurface stresses for line contact [33].

Both of the above models do not take into account the friction effects between rolling elements. It was confirmed [18,23,28] that in rolling bearings, the friction effects are very small, and practically do not affect the subsurface stress distributions.

Determination of the subsurface stress distribution requires the execution of the following calculation steps:

1. determination of the geometries of the rolling elements (the radii of curvatures, the contact length, etc.) and loading conditions,
2. determination of the material properties (static: the Young modulus—*E*, the Poisson’s ratio—ν; fatigue: the Wöhler diagrams for alternate bending and alternate torsion fatigue strength), which are discussed in chapter 3,
3. calculation of the loading acting on rolling elements—see Section 5.1,
4. calculation of the maximal contact stress *p_o_* and dimensions of the contact area (elliptical: semi-axes of the contact area; line: contact length)—see Section 5.2,
5. calculation of subsurface stresses using analytical formula—see Section 5.2,
6. estimation of fatigue loading or fatigue life (in cycles)—see Section 5.3.

### 5.1. Determination of Loading in Rolling Elements

The calculation method for the maximal force acting on rolling element *Q_max_* depends on the type of bearing (radial, angular, thrust) and direction of loading (radial—*F_r_*, axial—*F_a_*). The maximal loadings in rolling elements for various types of rolling bearings can be calculated as follows:

- thrust roller bearing and thrust ball bearing subjected to axial loading:
  (6) $$Q_{\max}=\frac{F_{a}}{i\cdot Z\cdot \sin(\alpha )},$$
- thrust roller bearing subjected to eccentric axial loading:
  (7) $$Q_{\max}=\frac{F_{a}\cdot A_{t\left(\mathrm{line}\right)}}{Z\cdot \sin(\alpha )},$$
- thrust ball bearing subjected to eccentric axial loading:
  (8) $$Q_{\max}=\frac{F_{a}\cdot A_{t\left(\mathrm{point}\right)}}{Z\cdot \sin(\alpha )},$$
- one-row radial/angular roller bearing subjected to radial loading:
  (9) $$Q_{\max}=\frac{F_{r}\cdot A_{n\left(\mathrm{line}\right)}}{i\cdot Z\cdot \cos(\alpha )},$$
- one-row radial/angular roller bearing subjected to radial and axial loadings:
  (10) $$Q_{\max}=\max\left\{\frac{F_{r}\cdot A_{n\left(\mathrm{line}\right)}}{Z\cdot \cos(\alpha )};\;\frac{F_{a}\cdot A_{t\left(\mathrm{line}\right)}}{Z\cdot \cos(\alpha )}\right\},$$
- one-row radial/angular ball bearing subjected to radial loading:
  (11) $$Q_{\max}=\frac{F_{r}\cdot A_{n\left(\mathrm{point}\right)}}{i\cdot Z\cdot \cos(\alpha )},$$
- one-row radial/angular ball bearing subjected to radial and axial loadings:
  (12) $$Q_{\max}=\max\left\{\frac{F_{r}\cdot A_{n\left(\mathrm{point}\right)}}{Z\cdot \cos(\alpha )};\;\frac{F_{a}\cdot A_{t\left(\mathrm{point}\right)}}{Z\cdot \cos(\alpha )}\right\},$$

The prediction of fatigue life or admissible fatigue load for the rolling bearing is based on the knowledge about the forces operating on rolling elements. The distribution of forces over rolling elements and their maximum values are the main interest. The formula for the force calculations mainly depends on the type of rolling bearing and the space orientation of forces imposed on the rolling bearing. The simplest case appears for the thrust roller or ball bearings, subjected only to axial loads (6). For such bearings, all of the rolling elements are equally loaded in each moment of operation. Such conclusion in a meaning way simplifies the calculations of admissible parameters, such as maximum fatigue load and fatigue life expectancy. In both cases, *i* stands for the number of rows of rolling elements, *Z* stands for the number of rolling elements in one row, α means the angle of bearing operation, and *F_a_* is the external axial load. This ideal situation of operation appears in a rather limited area of engineering applications. In the case of the spatial systems of loads, more complex formulas are demanded. Here, the starting point is the shape of the contact area and forces, which can be calculated for the respective case. In general, the dependency between the force and the deflection in rolling bearing takes the below form [51]:

(13) $$F=K_{j}\cdot \delta_{defl}{}^{j},$$

where *j* = 1.5 for ball bearings, and is equal to 1.0 for roller bearings. *K_j_* depends only on the geometry of the contact and material properties, so that *K_j_* can be considered generalized stiffness. The problem becomes even more complex when one takes into consideration the analysis of deflections and influence of clearances, which exist in the analyzed bearing. Formulas for the maximum load appearing in the rolling elements are depicted in Equations (7)–(12). In these formulas (7)–(12), the coefficients of maximal load for the most heavily charged rolling element—*A_n(line)_*, *A_n(point)_*, *A_t(line)_* or *A_t(point)_*—are included. The expressions for the above coefficients are the elliptic integrals, and depend on the angle of the load distribution:

(14) $$J_{n}=A_{n}{}^{-1}=\frac{1}{2\pi }\int_{-\psi_{\varepsilon }}^{\psi_{\varepsilon }}{\left[1-\frac{1}{2\varepsilon }\left(1-\cos\left(\psi \right)\right)\right]}^{n_{\varepsilon }}\cos\left(\psi \right)d\psi ,$$

(15) $$J_{t}=A_{t}{}^{-1}=\frac{1}{2\pi }\int_{-\psi_{\varepsilon }}^{\psi_{\varepsilon }}{\left[1-\frac{1}{2\varepsilon }\left(1-\cos\left(\psi \right)\right)\right]}^{n_{\varepsilon }}d\psi ,$$

where $\varepsilon =0.5[1-\cos(\psi_{\varepsilon })]$ and exponent *n*_ε_ is set to 3/2 for point contact, 10/9 for linear contact, or 4/3 for combined contact: linear in one raceway, and a point on the second raceway. Here, *ψ_ε_* is the angle corresponding to the effectively loaded part of the bearing raceway circumference.

**Figure 6:**
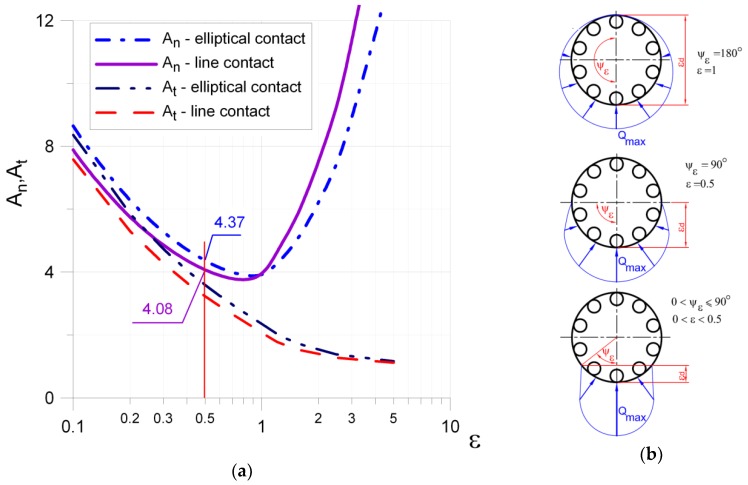
Influence of angle of load distribution *ψ_ε_* on *A* coefficients: (**a**) The values of the coefficients *A_n_* and *A_t_* of the maximal loading for line and point contact; (**b**) Load distribution on rolling elements in radial bearing angle corresponding to the effectively loaded part.

The exemplary values of these *A* coefficients for the line and point contact (radial bearing) are shown in Figure 6; these can be also found in Ref. [51].

If we take into consideration the radial, roller, or ball bearings subjected to radial force, and the bearing is assembled with the common clearance suggested by the manufacturers, the real angle *ψ_ε_* of the loaded rolling elements varies between 70–90°. In such situations, the formula for the maximal load of the most strenuous rolling element significantly simplifies, and can be approximated with the same formula for both types of bearings:

(16) $$Q_{\max}=\frac{5\cdot F_{r}}{i\cdot Z}.$$

Such a formula is a conservative approximation of the maximal load, and was derived by Stribeck in 1901.

### 5.2. Determination of Contact Parameters

The dimensions of the contact area and the maximal contact pressure will be presented with respect to the line and elliptical contact. In the case of the line contact (i.e., contact between two long parallel cylinders), the rectangular contact area can be described as one-half the width of the contact strip:

(17) $$b=\sqrt{\frac{4P^{*}\cdot R^{*}}{\pi \cdot E^{*}}},$$

where P* is the radial loading per unit length, and:

(18) $$R^{*}={\left(1/R_{11}+1/R_{21}\right)}^{-1},$$

(19) $$E^{*}={\left(\frac{1-\nu_{1}{}^{2}}{E_{1}}+\frac{1-\nu_{2}{}^{2}}{E_{2}}\right)}^{-1}.$$

The maximal contact pressure is equal to:

(20) $$p_{o}=\sqrt{\frac{P^{*}\cdot E^{*}}{\pi \cdot R^{*}}}.$$

For the elliptical contact, the semi-axes *a* and *b* and the maximal contact pressure *p_o_* can be calculated using given below formula:

(21) $$a=m\cdot \sqrt[{3}]{\frac{3\pi }{4}\frac{Q_{\max}\left(k_{1}+k_{2}\right)}{A+B},}$$

(22) $$b=n\cdot \sqrt[{3}]{\frac{3\pi }{4}\frac{Q_{\max}\left(k_{1}+k_{2}\right)}{A+B}},$$

(23) $$p_{o}=\frac{3}{2}\frac{Q_{\max}}{\pi \cdot a\cdot b},$$

where:

(24) $$k_{i}=\frac{1-\upsilon_{i}^{2}}{\pi \cdot E_{i}};\;i=1,\;2.$$

The Hertz coefficients *m* and *n* were determined by Timoshenko in the range θ∈(30°, 90°) with a step of 5° [52] and by Cooper in the range θ∈(1°, 90°) with a step of 1° [53]. On the basis of these results, the authors proposed the continuous functions of the auxiliary *m* and *n* coefficients. These functions are very useful for numerical calculations, and are as follows:

(25) $$m^{-1}=-0.072576\cdot \theta^{4}+0.306757\cdot \theta^{3}-0.425848\cdot \theta^{2}+0.817353\cdot \theta +0.018040,$$

(26) $$\begin{array}{l} n=-0.640562\cdot \theta^{6}+3.471455\cdot \theta^{5}-7.405219\cdot \theta^{4}+7.984778\cdot \theta^{3}-4.592703\cdot \theta^{2}+\\ +1.771294\cdot \theta +0.108768 \end{array}$$

**Figure 7:**
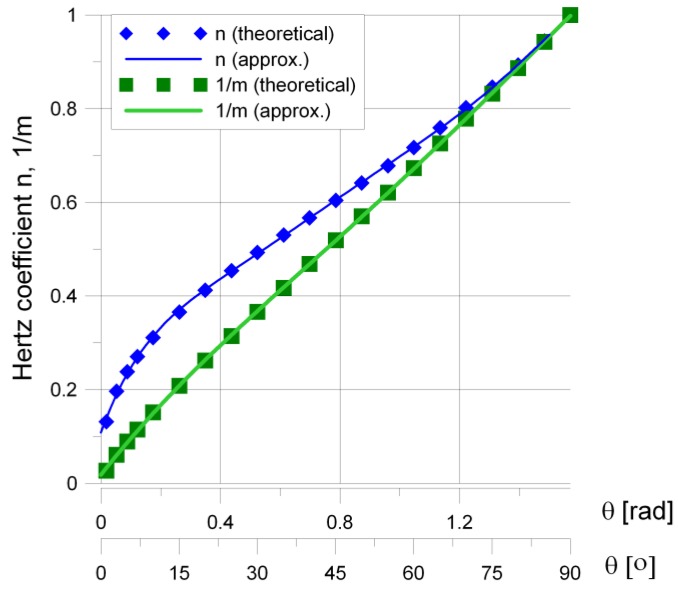
Distribution of *m* and *n* parameters in the function of angle θ.

The distributions of *m* and *n* as functions of auxiliary angle θ are also presented in Figure 7. Both of the above functions are valid for θ∈(1°, 90°).

The auxiliary angle θ can be calculated as follows:

(27) $$\theta =\cos^{-1}\left(\frac{B-A}{A+B}\right),$$

where the constants *A* and *B* can be calculated from the given below formula:

(28) $$A+B=0,5\left(R_{11}^{-1}+R_{12}^{-1}+R_{21}^{-1}+R_{22}^{-1}\right),$$

(29) $$B-A=0,5\cdot \sqrt{{\left(R_{11}^{-1}+R_{12}^{-1}\right)}^{2}+{\left(R_{21}^{-1}+R_{22}^{-1}\right)}^{2}+2\left(R_{11}^{-1}+R_{12}^{-1}\right)\left(R_{21}^{-1}+R_{22}^{-1}\right)\cos\left(2\varphi \right)},$$

where *R*_11_ and *R*_12_ are the minimum and the maximum principal radii of curvatures of the first body at the initial contact point, respectively, *R*_21_ and *R*_22_ are the corresponding values of radii for the second body, and *φ* is the angle between the planes of the principal curvatures of the two surfaces.

The subsurface stresses in the respective rolling bearing elements can be calculated using the theoretical solutions proposed in the literature:

- the analytical model for circular and elliptical contact proposed by Sackfield and Hills [32] for the calculation of ball bearings,
- the solution for line contact given by Radzimovsky [33] for roller bearings.

Both models are based on the Hertz approach, and are presented in detail in Refs [32,33] and also verified in Refs [18,23]. Due to the complexity and the number of respective equations required for the calculations of the subsurface stress distributions and the availability of both solutions in the working literature, the particular formulas are not included in the present paper.

### 5.3. Application of Fatigue Life Model

Different probabilistic engineering and deterministic research fatigue models for the prediction of the fatigue life of rolling bearings were proposed. Sadeghi et al. provided a detailed description and revision of such models [4]. The main aim of the present study is to propose the procedure of fatigue strength/life prediction based on the MHCF criteria. The application of such an approach supplemented with the theoretical formula for subsurface stress distributions (for line and elliptical contact) enables the prediction of the fatigue life/strength of rolling bearings, as determined by the geometry of the rolling elements and the loading conditions. It should be noted that the proposed approach does not require expensive and time-consuming experimental tests for rolling bearings, which are necessary for classical engineering models.

**Figure 8:**
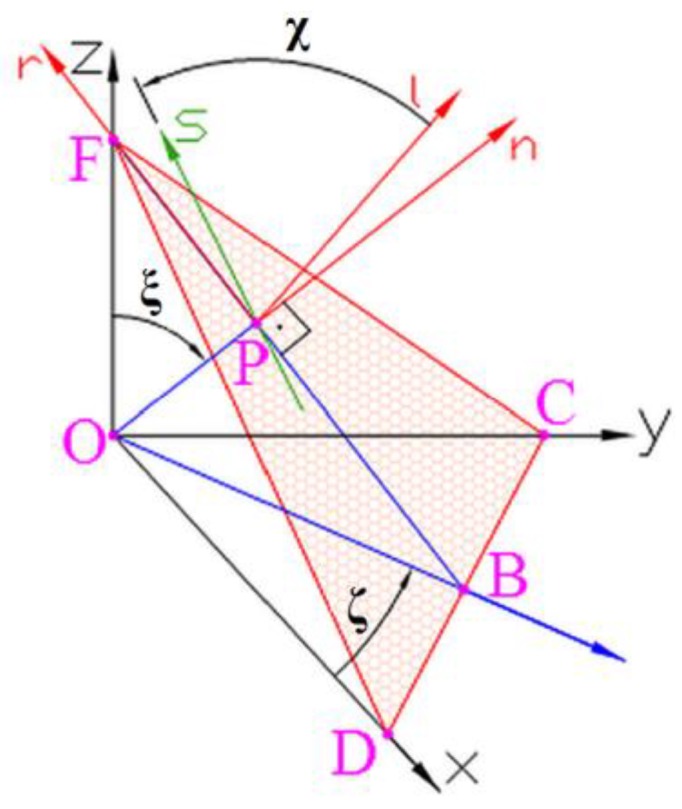
Orientation of material plane Δ(CDF) crossing point O (point P tends to point O); angle χ defines the direction of versos **s** on material plane Δ; versor **n** is perpendicular to material plane Δ.

A promising alternative for the fatigue life estimation of rolling bearings is an application of the multiaxial high-cycle fatigue criteria (MHCFC). Such hypotheses take into account the complex distribution of multiaxial and non-proportional stresses in time on the equivalent fatigue level. The MHCF model should take into account the multiaxial stress state with tri-axial compressive stresses, as well as the shift in-phase between the normal and shear stresses on the fatigue damage of high hardness bearing steels. The verification of MHCFC for RCF problems was discussed in Ref [28]. The best agreement with experimental tests was obtained for Papadopoulos MHCFC based on the integral approach (the model was proposed in 1997) [30]. This criterion (marked as P_1_) takes into account the volumetric root mean square of shear stress τ_a_ and the maximal value of hydrostatic stress σ_H,MAX_ as follows:

(30) $$\tau_{P1}=\frac{1}{k_{s}}\sqrt{\frac{5}{8\pi^{2}}\int_{\zeta =0}^{2\pi }\int_{\xi =0}^{\pi }\int_{\chi =0}^{2\pi }\tau_{a}^{2}\left(\zeta ,\xi ,\chi \right)d\chi \cdot \sin\left(\xi \right)\;d\xi \;d\zeta }+\left(\frac{3\tau_{-1}}{\sigma_{-1}}-\sqrt{3}\right)\sigma_{H,\max}\leq \tau_{-1},$$

where:

(31) $$\tau_{a}\left(\zeta ,\xi ,\chi \right)=0.5\left[\underset{t\in T}{\max}\tau \left(\zeta ,\xi ,\chi ,t\right)-\underset{t\in T}{\min}\tau \left(\zeta ,\xi ,\chi ,t\right)\right],$$

and angles *ζ*, *ξ*, and χ are defined in Figure 8, and *k_s_* is the size factor.

**Figure 9:**
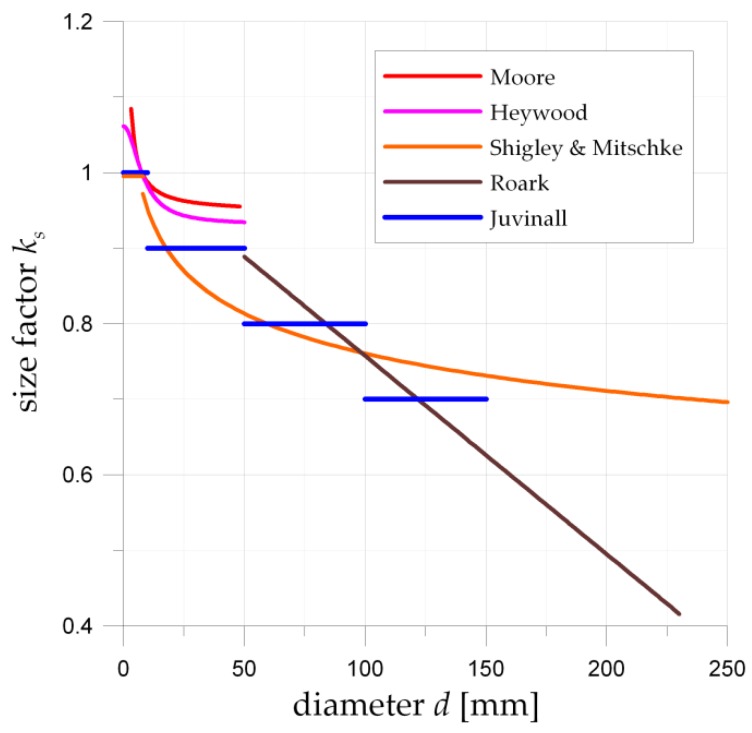
Graphical representation of size factors values *k_s_* for various diameters *d* of the rolling elements for the respective models given in Equations (32)–(36).

The detailed description of the P_1_ model is presented in Ref [29,30]. It should be noted that the size factor *k_s_* is not taken into account in the original P_1_ criterion. The size effect is also often omitted in fatigue calculations, which can lead to an overstatement of the safety factor of real structures. The impact of *k_s_* becomes more and more important as the size of the objects increases. Generally, the size factor is defined as a function of a cross-section area or equivalent diameter of an object [54]. The selected formulations of the size factor [54] are given below:

- Juvinall proposal
  (32) $$k_{s}=\{\begin{array}{c} 1\;\mathrm{for}\;d\leq \;10\;\mathrm{mm}\\ 0.9\;\mathrm{for}\;10<d\leq \;50\;\mathrm{mm}\\ 0.8\;\mathrm{for}\;50<d\leq \;100\;\mathrm{mm}\\ 0.7\;\mathrm{for}\;100<d\leq \;150\;\mathrm{mm} \end{array}$$
- Heywood model
  (33) $$k_{s}=0.931\left(1+\frac{9.032}{64.516+d^{2}}\right)\;\mathrm{for}\;d\leq \;50\;\mathrm{mm}$$
- Moore model
  (34) $$k_{s}=\frac{0.947}{1-0.406/d}\;\mathrm{for}\;3.2\leq d\leq \;50\;\mathrm{mm}$$
- Shighley and Mitschke model
  (35) $$k_{s}=\{\begin{array}{c} 1\;\mathrm{for}\;d\leq \;8\;\mathrm{mm}\\ 1.189d^{-0.097}\;\mathrm{for}\;8<d\leq \;250\;\mathrm{mm} \end{array}$$
- Roark model
  (36) $$k_{s}=1-\frac{d-7.62}{381}\;\mathrm{for}\;50\leq d\leq \;230\;\mathrm{mm}$$

and compared in Figure 9. In order to determine the effect of the size of the bearing elements, the equation proposed by Shigley and Mitschke (35) was selected. This model is continuous in the range 8 < *d* ≤ 250 mm, and is in good agreement with the definition proposed by Juvinall (32) and more conservative than the proposals of both Heywood (33) and Moore (34).

## 6. Results and Discussion

The detailed calculations were performed for several arbitrarily chosen roller and ball bearings (both thrust and radial). In all of the considered examples, the values of subsurface stresses were calculated by means of the analytical formulas proposed in Refs [32,33]. After that, these values were introduced to the considered hypothesis of the multiaxial high cycle fatigue. Usage of this hypothesis enabled an assessment of the limiting value of the maximal fatigue load. In these calculations, the standard criterion with 90% damage-free bearings after one million rolling-bearing revolutions was utilized. It is worth emphasizing that only the knowledge about geometrical characteristics, such as the dimensions of rolling elements, raceways radii, and number of rolling elements are needed in the proposed approach. The only material parameters that are needed in such calculations are the values of the fatigue properties that are received in standard fatigue tests for the assumed number of cycles (see chapter 3).

**Figure 10:**
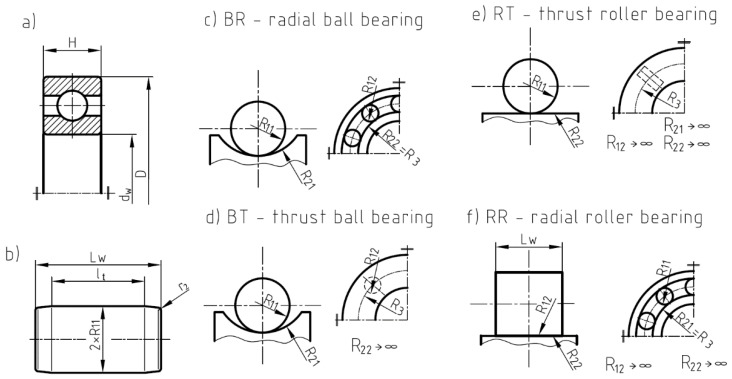
Dimensions of rolling elements and definitions of principal radii of curvatures in rolling bearings: (**a**) principal dimensions of rolling bearing, (**b**) dimensions of rollers, (**c**) principal radii of curvatures in radial ball bearing (BR), (**d**) principal radii of curvatures in thrust ball bearing (BT), (**e**) principal radii of curvatures in thrust roller bearing (RT), (**f**) principal radii of curvatures in radial roller bearing (RR).

The catalogue data for the considered roller and ball bearings are given in Table 2 and Table 3, respectively. The detailed values for investigated rolling bearings were taken from the SKF catalogue [55]. Certain information was additionally identified on the base of technical drawings accessible by the manufacturer [55]. In these tables, the following information were included (details in Figure 10): the designation of the rolling bearings and the assumed parameters of the contact area between elements (*f_L_*, *f*), the principal dimensions (*d*—internal diameter, *D*—external diameter, *H*—bearing width), the radius of the principal curvatures of the roller or ball (*R*_11_ and *R*_12_), the effective and the total length of the roller (*l_t_* and *L_w_*, respectively), the radius on which the roller and ball elements are rolling (*R*_3_), the principal radii of the raceway (*R*_21_ and *R*_22_), the number of rolling elements (*Z*), the catalogue fatigue load (*F_u_^CAT^*), the calculated maximal fatigue load (*F_u_^P1^*), and difference δ. The *f_L_* is the parameter that defines the effective length of a roller, and *f* is the rate conformity ratio for the ball bearings (see Equation (5)). The effective lengths of rollers are calculated using the following formula:

(37) $$l_{t}=f_{L}\cdot L_{W}-2r_{2}.$$

In both tables, the results of the analytical calculations are given. These results were obtained with the use of the Papadopoulos *F_u_^(P1)^* criterion (30).

In the case of the roller bearings, all of the calculations were made under the assumption that the effective lengths of the contact line *l_t_* (see Figure 5) varied within the range: *f_L_* = 80–100% of the total length *L_w_* of the roller reduced by the end fillets. The values of the fillets were equal to the mean value of the respective minimum and maximum values given in the standards [48]. Therefore, such an approach takes into account the real shape of the rollers, which are at least chamfered or smoothly rounded at the ends of the roller in order to eliminate the stress peaks at the ends of the rollers (see Figure 5). Zaretsky et al. discussed the different shapes of the rollers and the effects of the edge contact stresses [16,17].

In the case of ball bearings, the relation between the radius of the ball and the radius of the raceway has a crucial influence on the stress level. This relationship is defined by means of the coefficient of the race conformity ratio *f* (5). Such coefficients are not given in bearing catalogues. The values of *f* = 0.51–0.54 are the most frequently used in practice [51]. The calculations were made for this value and for the arbitrary chosen thrust and radial ball bearings (see Table 3).

For the radial bearing, it was also assumed that half of the bearing is effectively loaded (which means that the load angle ψ_ε_ = 90°, so that ε = 0.5). The corresponding load coefficients (see Figure 6) are equal to *A_n_* = 4.37 (for radial ball bearings) and *A_n_* = 4.08 (for radial roller bearings).

The difference between the calculated fatigue load *F_u_^P1^* and the catalogue fatigue load *F_u_^CAT^* was calculated as below:

(38) $$\delta =\frac{F_{u}^{CAT}-F_{u}^{P1}}{F_{u}^{CAT}}\cdot 100\%.$$

The number of cycles *N*_1_ per one full turn of the bearing was calculated as follows:

- for the thrust ball and roller bearing:
  (39) $$N_{1}=\{\begin{array}{c} 2\frac{R_{11}}{R_{3}}\;\mathrm{for}\;\mathrm{rolling}\;\mathrm{element}\\ Z\;\mathrm{for}\;\mathrm{raceway}\; \end{array},$$
- for the radial ball and roller bearing:
  (40) $$N_{1}=Z\;\mathrm{for}\;\mathrm{raceway}.$$

In the case of the radial bearings, fatigue calculations were only made for the raceways. It is justified by the number of fatigue cycles in the rolling element per one turn (in assumed working conditions: ε = 0.5) of the bearing is much lower than the number of cycles in the raceway. This effect can be observed for the NU 2304 ECP bearing in Table 2. In this example, the number of fatigue cycles in the roller is equal to *N*_1_ = 3.1, but for the inner raceway, *N*_1_ = 5 (for the rotating inner raceway, ε = 0.5) or *N*_1_ = 10 (for the rotating outer raceway). Due to these observations, the maximal fatigue loads for all of the radial bearings were only presented for the most disadvantageous loading conditions (rotating outer raceway; fatigue occurs in the inner raceway).

In all of the considered cases, very good agreement is observed between the calculated and the catalogue (SKF) maximal fatigue loads. The absolute values of differences δ were smaller than |δ|≤10%. The negative difference δ means underestimation, and the positive difference δ means overestimation of the maximal fatigue load *F_u_^P1^* with respect to the catalogues one *F_u_^CAT^*.

In the case of the thrust and radial roller bearing, very good agreement of the predicted fatigue load *F_u_^P1^* with respect to *F_u_^CAT^* was obtained. The calculations were made for the following values *f_L_* = 80%, 90%, or 100% (this parameter is not given in catalogue data, and was assumed in calculations). Only the selected results (for *f_L_* for which |δ|≤10%) have been presented in Table 2 and Table 3. It should be emphasized that such very good agreement was obtained assuming the typical shape of the rollers (Table 2), in which the end parts are slightly rounded (in order to minimize the edge stress effect).

Very good agreement was also obtained in the case of the ball bearings. For the investigated bearings, the parameter *f* varying from 0.53 to 0.55, and 0.57 (in one case—bearing 53204) was used (this parameter is also not given in the catalogues). The parameter *f*, which was used in the above calculations, remains in agreement with the values recommended in the literature [51]. It was also observed that the raceways of the rolling bearing are more exposed to fatigue failure. This applies to all the thrust and radial roller bearings (Table 2) as well as the thrust and radial ball bearings (Table 3).

It is worth mentioning that the numerical results, which follow the proposed in the paper procedure, do not call for the knowledge of catalog admissible values for the dynamic, static, or fatigue loads. The material and geometrical properties were needed in all of the calculations of the studied bearings. This means that the maximum load for the considered bearing can be assessed without time-consuming and expensive experimental tests. The proposed approach can also substitute the time-consuming FEM calculations (in case of contact analysis, a large number of finite elements in the area of contact zone is needed) by means of simpler methods, which provides a quick and precise assessment of the admissible load for the analyzed bearing.

## 7. Conclusions

The new methodology for the fatigue life prediction of the rolling bearing is proposed and verified in the paper. The proposed method is based on the application of the multiaxial high-cycle fatigue criterion and the analytical solutions for elliptical and line contact for the estimation of the equivalent fatigue stress in the rolling bearing. It should be also mentioned that the size effect, which is generally not included in the fatigue criteria, was implemented in the adopted fatigue model. Taking into account both the effect of size and non-proportional multiaxial stress state with high compression stresses results in a very good agreement of the predicted maximal fatigue load with corresponding values given in the bearing catalogues. The verification of the proposed methodology was made for both the ball and roller bearing (thrust and radial) in a wide range of diameters (*d_w_* = 15–710 mm). In all of the investigated (randomly selected) examples, the differences between the predicted and catalogue fatigue loads were smaller than 10%. However, it should be noted that not all of the information (concerning bearing geometry) has been given in catalogues; however, the values of the parameters that are assumed in calculation were within the typical ranges for rolling bearings (*f_L_* = 80–100%, *f* = 0.52–0.57).

Summarizing, the proposed methodology gives the following advantages:

1. Only the geometries of bearing and rolling elements and two Wöhler curves for the material (*τ*_−1_ and *σ*_−1_) are necessary for the analysis,
2. The method does not need the additional data from experimental tests for an investigated rolling bearing,
3. The calculations can be made quickly (using analytical solutions), including real multiaxial stress state in elements,
4. The obtained results are consistent with the data given in catalogues.

Due to this, the method can be used for fatigue life prediction, calculation of the maximal fatigue load, and also optimization of the shapes of the rolling elements of rolling bearings. The methodology presented in the paper can be also used for other applications in which contact occurs, such as, rail–wheel contact, cam–gear contact, gear teeth, etc.

## Figures and Tables

**Table 1:** Chemical composition (in wts %) of AISI 52100 bearing steel.

Chemical Composition (wts) [%]	C	Cr	Si	Mn	P	S
AISI 52100	0.95–1.05	1.30–1.65	0.15–0.35	0.25–0.45	≤0.027	≤0.025

**Table 2:** Results of fatigue life calculations for roller bearings under an *N*_10%_ reliability condition concerning one million bearing rotations.

Designation and Type ^1^ and *An* and *f_L_*	Principal Dimensions, Rollers Dimensions, Principal Radii of Curvatures, Number of Rolling Elements, Catalogue Fatigue Load [55]						Calculated Maximal Fatigue Loading		Difference	
	*d_w_/D/H* mm	*R*_11_ mm	*l_t_* (*L_w_*) mm	2*R_3_ mm	*Z*	*F_u_^CAT^* kN	*F_u_^P1^* kN		δ %	
							Roller	Ring	Roller	Ring
K81102TN, RT, *f_L_* = 80%	15/28/9	1.75	1.9 (3.5)	21.5	12	2.45	2.48	2.49	1.2	1.6
81104TN, RT, *f_L_* = 80%	20/35/10	2.25	2.7 (4.5)	27.87	13	4.65	4.9	4.84	5.4	4.1
K81206TN, RT, *f_L_* = 80%	30/52/16	3.75	5.1 (7.5)	41.5	12	13.4	14.54	14.28	8.5	6.6
81106TN, RT, *f_L_* = 80%	30/47/11	2.5	3.1 (5)	38.82	17	7.65	7.82	7.65	2.2	0
K81110TN, RT, *f_L_* = 90%	50/70/14	3	4.5 (6.0)	59.5	24	16.6	18.19	17.52	9.6	5.5
K81113TN, RT, *f_L_* = 90%	65/90/18	3.75	5.85 (7.5)	78	29	32.5	35.53	33.21	9.3	2.2
K89413TN, RT, *f_L_* = 100%	65/140/15	7.5	28.4 (30)	103	15	143	162.5	144.5	13.6	1.0
K81224TN, RT, *f_L_* = 100%	120/170/39	7.5	13.4 (15)	145	24	104	114	109.1	9.6	4.9
81240M, RT, *f_L_* = 90%	200/280/62	13	21.6 (26)	246.5	22	255	262.2	255.2	2.8	0.1
891/710M, RT, *f_L_* = 80%	710/850/85	18	26.4 (36)	780	56	900	918.4	868	2.04	−3.6
NU2304ECP, RR, *An* = 4.08, *f_L_* = 80%	20/52/21	4.5	9.9 (14)	27.5	10	4.8	6.3 (*N*_1_ = 3.1)	(a) 4.9 (*N*_1_ = 10) (b) 5.69 (*N*_1_ = 5)	31.3	(a) 2.1 (b) 18.5
NU410, RR, *An* = 4.08, *f_L_* = 100%	50/130/31	10	18.8 (20)	70.8	10	16.6	-	17.94	-	8.1
NJ220ECP, RR, *An* = 4.08, *f_L_* = 100%	100/180/34	11	22.2 (24)	119	17	36.5	-	38.54	-	5.6
NJ340ECML, RR, *An* = 4.08, *f_L_* = 90%	200/420/80	28	47.4 (56)	258	15	150	-	157.7	-	5.1
NUP19/600ECMA/HA1, RR, *An* = 4.08, *f_L_* = 80%	600/800/90	27	40.2 (54)	649	35	275	-	280.5	-	2

^1^ RT—Thrust roller bearing, RR—radial roller bearing

**Table 3:** Results of fatigue life calculations for ball bearings under an *N*_10%_ reliability condition concerning one million bearing rotations.

Designation and Type ^2^ and *An* and *f*	Principal Dimensions, Rolling Element Dimensions, Principal Radii of Curvatures, Number of Rolling Elements, Catalogue Fatigue Load [55]						Calculated Maximal Fatigue Loading		Difference	
	*d_w_/D/H* mm	*R*_11_ = *R*_12_ mm	*R*_21_ mm	2*R_3_ mm	*Z*	*F_u_^CAT^* kN	*F_u_^P1^* kN		δ %	
							r.e. ^2^	Ring	r.e. ^2^	Ring
53204, BT, *f* = 0.57	20/40/14.7	3.57	−4.07	32	12	1.4	1.6	1.45	14.3	3.6
53307, BT, *f* = 0.55	35/68/25.6	6	−6.6	52	10	3.55	3.73	3.60	5.1	1.4
53310, BT, *f* = 0.55	50/95/34.3	7.94	−8.734	72	11	6.3	6.54	6.22	3.81	−1.3
53316, BT, *f* = 0.54	80/140/47.6	11	−11.88	110	12	13.7	13.8	13.1	0.73	−4.4
53228, BT, *f* = 0.54	140/200/48.6	11	−11.88	170	19	17.6	19.0	18.0	8.0	2.3
6304, BR, *An* = 4.37, *f* = 0.54	20/52/15	4.765	−5.146	26.43	7	0.34	-	0.33	-	−2.0
6310, BR, *An* = 4.37, *f* = 0.53	50/110/27	9.53	−10.102	60.95	8	1.6	-	1.48	-	−7.5
6320, BR, *An* = 4.37, *f* = 0.53	100/215/47	18.26	−19.356	121.0	8	4.75	-	4.49	-	−5.5
6040, BR, *An* = 4.37, *f* = 0.53	200/3310/51	16.67	−17.67	221.7	14	6.4	-	6.7	-	4.7
6080M, BR, *An* = 4.37, *f* = 0.54	400/600/90	30.16	−32.573	439.7	15	16.3	-	16.1	-	−1.2

^2^ BT—Thrust ball bearing, BR—radial ball bearing, r.e.—rolling element

## Nomenclature

*a*_1_—modification factor for reliability
*a*_2_—material and processing factor
*a*_3_—application factor
*A_n_, A_t_*—coefficient of the maximal loading for line and point contact
*a*, *b*—semi-axes of contact area or contact length of rectangular contact area
*C*—basic dynamic load rating [N]
*d*—diameter of rolling element
*d_w_*—bore diameter of bearing
*D*—outside diameter of bearing
*E**—equivalent Young’s modulus
*f*—race conformity ratio
*f_L_*—coefficient which defines the effective length of roller
*F_a_*—applied axial loading
*F_r_*—applied radial loading
*F_u_^CAT^*—catalogue fatigue load
*F_u_^P1^*—calculated fatigue load
*H*—bearing width
*i*—number of rows of rolling elements
*k*—exponent of bearing type
*k_s_*—size factor
*K_j_*—generalized stiffness
*L*—rated service life (in millions of revolutions) for 10% probability of failure
*L_w_*—roller length
*l_c_*—crowned length of roller
*l_t_*—flat length of roller
*m, n, A, B*—coefficients of elliptical contact
*N*—number of cycles to failure
*N*_1_—number of cycles per one full turn of bearing
*P*—external equivalent load [N]
*P**—radial loading per unit length
*p_o_*—maximal contact pressure
*Q_max_*—maximal load of the most strenuous rolling element
*R**—equivalent radius
*R*_11__,_*R*_12_—minimum and the maximum principal radii of curvatures of the first body at the initial contact point, respectively
*R*_21_, *R*_22_—minimum and the maximum principal radii for the second body at the initial contact point, respectively
*R*_3_—radius on which the rolling elements are rolling
*r*_2_—fillet radius of the edges of roller
*t*—time
*T*—time of one cycle
*Z*—number of rolling elements in one row
α—angle of bearing operation
ε — coefficient of loading distribution on rolling element
δ—difference between the calculated and catalogue fatigue load
δ_defl_—total deflection in rolling bearing
τ—shear stress
τ_a_—amplitude of shear stress
ζ, ξ, χ—angles defining material plane and direction of shear stress on material plane
*φ*—angle between the planes of principal curvatures of the two surfaces
θ—auxiliary angle
σ—normal stress
σ_H,MAX_—maximal hydrostatic stress
σ_VM_—Von Mises stress
σ_−1_—alternate bending fatigue strength
τ_−1_—alternate torsion fatigue strength
τ_P1_—equivalent fatigue stress
ν—Poisson’s ratio
*ψ*—location angle
*ψ_ε_*—angle corresponding to the effectively loaded part of the bearing raceway circumference
